# Activity-Based Labeling of Matrix Metalloproteinases in Living Vertebrate Embryos

**DOI:** 10.1371/journal.pone.0043434

**Published:** 2012-08-28

**Authors:** Jonathan Y. Keow, Eric D. Pond, Justin S. Cisar, Benjamin F. Cravatt, Bryan D. Crawford

**Affiliations:** 1 Department of Biology, University of New Brunswick, Fredericton, New Brunswick, Canada; 2 Department of Chemical Physiology, The Scripps Research Institute, La Jolla, California, United States of America; Stanford University, United States of America

## Abstract

Extracellular matrix (ECM) remodeling is a physiologically and developmentally essential process mediated by a family of zinc-dependent extracellular proteases called matrix metalloproteinases (MMPs). In addition to complex transcriptional control, MMPs are subject to extensive post-translational regulation. Because of this, classical biochemical, molecular and histological techniques that detect the expression of specific gene products provide useful but limited data regarding the biologically relevant activity of MMPs. Using benzophenone-bearing hydroxamate-based probes that interact with the catalytic zinc ion in MMPs, active proteases can be covalently ‘tagged’ by UV cross-linking. This approach has been successfully used to tag MMP-2 *in vitro* in tissue culture supernatants, and we show here that this probe tags proteins with mobilities consistent with known MMPs and detectable gelatinolytic activity in homogenates of zebrafish embryos. Furthermore, because of the transparency of the zebrafish embryo, UV-photocroslinking can be accomplished *in vivo*, and rhodamated benzophenone probe is detected in striking spatial patterns consistent with known distributions of active matrix remodeling in embryos. Finally, in metamorphosing *Xenopus* tadpoles, this probe can be used to biotinylate active MMP-2 by injecting it and cross-linking it *in vivo*, allowing the protein to be subsequently extracted and biochemically identified.

## Introduction

Embryonic morphogenesis, wound healing, and many pathological processes such as tumor metastasis involve cellular processes that are integrated and exquisitely regulated at multiple levels, many of which remain poorly understood. Among these, the dynamics of extracellular matrix (ECM) remodeling has been the focus of intense investigation for many years. The matrix metalloproteinases (MMPs) are zinc-dependent endopeptidases best known for their ability to hydrolyze ECM components [2], [3], and they have been recently recognized for their activities on other extracellular and intracellular substrates [4], [5]. Mis-regulation of these proteases is central to the pathologies of arthritis, tumor metastasis, various cardiovascular disorders and many other diseases [6]–[9]. Recently, the importance of regulated MMP activity in learning and memory has begun to emerge, implicating these proteases in the mechanisms of higher cognitive activity, and their mis-regulation in the devastating diseases that impair it [10]–[13].

Over the past four decades, MMP research has employed an enormous diversity of biochemical, molecular, cell-biological and high-throughput techniques. However, it has become increasingly apparent that, because of the complexity of the post-translational regulation of MMPs, assays that elucidate the activity of these enzymes, especially *in vivo*, are essential to the development of a coherent understanding of the dynamics of ECM remodeling [14], [15]. Recently, approaches to this problem using FRET-quenched substrates have been employed in tissue culture [16], [17] and even in whole animal models [18]–[21]. However, while these assays provide sensitive and potentially high-resolution data regarding the activity of MMPs, they do not directly address the identity of the active proteases, and represent a challenge for quantitative analysis.

**Figure 1:**
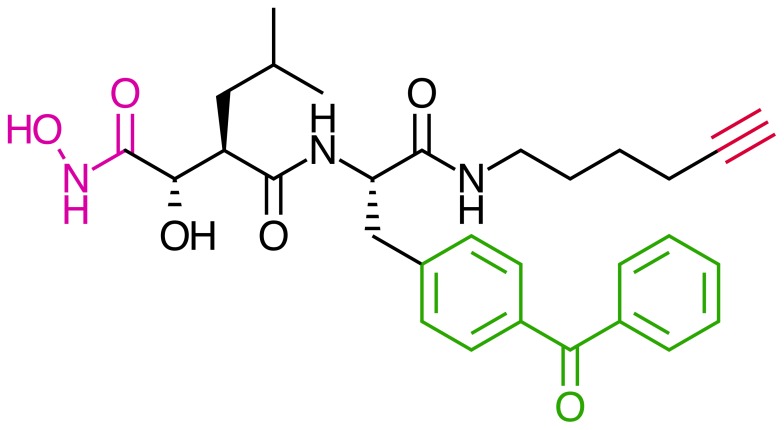
Structure of the Hx-PByne probe. The MMP labeling probes used were versions of this structure (compound 4 in [1]). The hydroxamic acid moiety (pink) chelates the catalytic zinc ion, and the benzophenone moiety (green) functions as a UV-photoactivateable crosslinking group. The terminal alkyne (red) allows the addition of groups such as biotin or rhodamine by click chemistry [1].

Activity-based protein profiling (ABPP) uses a chemical probe that targets active MMPs and becomes covalently bound to the protease. This technique provides quantitative information about the abundance of active proteases, as well as qualitative information about their location and, potentially, their molecular identity. We have used a hydroxamate-benzophenone probe (HxBP) that has previously been demonstrated to specifically target active MMPs *in vitro* [1] (Fig. 1). Upon UV excitation, the benzophenone moiety forms a high-energy species, which can insert into close proximity residues [22] allowing it to become covalently cross-linked to the active site of the enzyme.

We show that this probe can be used to label active MMPs in complex mixtures both *in vitro*, using homogenates of zebrafish embryos, *in vivo* by injecting the probe interstitially and cross-linking it *in situ*, then detecting the bound probe by confocal microscopy or identifying the labeled proteins using gelatin zymography and immunoblotting. The three and four dimensional patterns of MMP activity detected using this approach are consistent with previously published data regarding the distribution of ECM remodeling activity during zebrafish embryogenesis, and imply that the protein distribution of specific MMPs (such as Mmp2 in the zebrafish embryo) can give misleading impressions of ECM catabolism during embryogenesis. We propose that this *in vivo* ABPP presents a novel and potentially powerful tool to be added to the arsenal of techniques available to the research community investigating the dynamics of ECM remodeling *in living organisms*.

## Methods

### Animal Care and Husbandry

This study was carried out under the auspices of the University of New Brunswick Animal Care committee, and all animal used was governed by protocols in strict accordance with the recommendations of the Canadian Council for Animal Care. Zebrafish and *Xenopus* work was conducted under permits issued to BDC approved by the UNB Animal Care committee (UNB Animal care protocol numbers 10013 and 10016 respectively).

Sexually mature *Xenopus laevis* adults were obtained from Boreal Laboratories. Adults were kept in aquaria containing dechlorinated water at 22°C, and fed a standard diet. Adults were injected with 300 µl of human chorionic gonadotropin (500 i.u./ml) to induce mating, and eggs were collected and allowed to develop normally. Tadpoles were raised in dechlorinated water at 22°C and staged according to Nieuwkoop and Faber [23]. When tadpoles reached stage 50, triiodothyronine (T_3_) was added to a final concentration of 50 nM in the aquarium water, and induced tadpoles were collected after 0, 1, 2 or 3 days of exposure.

Zebrafish embryos were obtained by natural spawning of wild type fish maintained on a 14-hour light/10-hour dark cycle [24] and fed a diet of fish pellets, *Artemia* and *Drosophila* adults. After cleaning, sorting, and manual dechorionation using fine forceps, embryos were raised between 26 and 28.5°C in embryo rearing media (ERM) (13 mM NaCl, 0.5 mM KCl, 0.02 mM Na_2_HPO_4_, 0.04 mM KH_2_PO_4_, 1.3 mM CaCl_2_, 1.0 mM MgSO_4_, and 4.2 mM NaHCO_3_) and staged according to Kimmel *et al.* [25].

### Activity Profiling Probes Used

HxBP probes were synthesized as described in Saghatelian *et al*., 2004 in alkyne, rhodamine-conjugated, biotin-conjugated, and rhodamine-biotin conjugated forms. Stocks were diluted to 50 µM in DMSO, aliquoted and stored at −80°C until needed.

### *In vitro* Labeling of Human Recombinant MMP-2

Human recombinant MMP-2 was dissolved in activation buffer (50 mM NaCl, 50 mM Tris, 0.005% Triton X-100, pH 7.5) to a final concentration of 1 µg/ml in the presence or absence of 1 mM *p*-aminophenylmercuric acetate (APMA) and incubated 30 min at 37°C. HxBP (30 µM final concentration) or DMSO (vehicle control) was then added, and the solution was cross-linked using 48 mJ/cm^2^ of UV irradiation (Stratalinker 1800) (control tubes were left unexposed). The samples were mixed 1∶1 in reducing SDS-PAGE sample buffer (225 mM Tris pH 6.8, 50% glycerol, 5% SDS, 5% β-mercaptoethanol, 0.05% bromophenol blue).

20 µl of each sample was resolved on a 12% polyacrylamide gel for 2 hours at 120 V then horizontally transferred onto an Immobilon-P (Millipore) transfer membrane at 70 V for 2 hours. The blot was incubated in blocking buffer (5% BSA in phosphate-buffered saline with 0.01% Tween-20 (PBSTw)) overnight at 4°C to prevent nonspecific binding, and then incubated in Streptavidin-HRP conjugate (Invitrogen) (diluted 1∶10000 in blocking buffer) overnight at 4°C. The blot was then washed three times for 15 minutes in PBSTw to remove unbound streptavidin-HRP and detection of biotinylated proteins was performed using an enhanced chemiluminescence (ECL) kit (Pierce) according to the manufacturer’s directions.

### *In vitro* Labeling of MMPs from Whole Embryo Lysates

Hatched zebrafish embryos and larvae between 96 hpf to 168 hpf were terminally anesthetized in 0.4 mg/ml carbonate buffered tricaine and homogenized in 10 µl lysis buffer (150 mM NaCl, 10 mM HEPES pH 7.5, 10 mM CaCl_2_, 0.1% Triton X-100, 2× Protease Inhibitor (Sigma-Aldrich) (a cocktail of 4-(2-aminoethyl)benzenesulfonyl fluoride (AEBSF), pepstatin A, E-64, bestatin, leupeptin and aprotinin protease inhibitors, but which notably lacks EDTA and other MMP inhibitors)) per embryo. To verify the probe’s MMP-specificity, some samples were pre-incubated with 2.5 µM of GM6001 (Chemicon) inhibitor. HxBP was added to a final concentration of 5 nM and incubated overnight at 4°C. Tubes were then cross-linked with 120.0 mJ/cm^2^ of UV irradiation (Stratalinker 1800) (some control tubes were not cross-linked).

Three volumes of acetone were added to precipitate proteins, followed by a 20 minute incubation on ice. Samples were spun at 21,000 g at room temperature for 10 minutes and the pellets washed in 300 mM guanidine hydrochloride in 95% ethanol with 2.5% glycerol thrice for 10 minutes to remove acetone. Pellets were then washed twice in 95% ethanol with 3.5% glycerol for another 10 minutes each to remove residual salts. Finally pellets were dissolved in 2× SDS-PAGE reducing sample buffer with 3 M urea (at a ratio of 7.5 µL per embryo) and centrifuged to remove insoluble debris before being resolved on a 10% polyacrylamide gel at 120 V for 2 hours and transferred to PVDF (Immobilon-P) at 60 V for 3 hours. After blocking in 10% Bovine Serum Albumin (BSA) overnight, the membrane was incubated in 1∶10000 Streptavidin-HRP for 2 hours, washed thrice in PBSTw and biotinylated proteins detected by ECL (Pierce).

### *In vivo* Labeling of MMPs in Whole Zebrafish Embryos

Embryos were anesthetized in 0.04 mg/mL buffered tricaine solution and mounted in 3% methylcellulose dissolved in embryo rearing media (ERM). Using a pressure-based microinjection apparatus (ASI), these embryos were injected with a 5 µM solution of rhodamine-tagged HxBP dissolved in distilled water. Embryos were injected in the head between the developing eyes, or in the trunk dorsal to the yolk extension, allowed to recover for 5 minutes in fresh ERM, and then cross-linked with either 48.0 or 72.0 mJ/cm^2^ of UV irradiation (Stratalinker 1800). Control embryos were either pre-injected with 0.01 mg/ml GM6001 (diluted from a or not cross-linked as indicated. Embryos were fixed in 4% paraformaldehyde in PBS at 4°C overnight. After three washes in phosphate-buffered saline with 0.1% Triton X-100 (PBSTx), embryos were mounted for viewing using a Leica SP-2 confocal microscope.

### *In vivo* Labeling of MMPs in Developing Tadpoles

*X. laevis* tadpoles were raised to stage 50 and either induced to begin metamorphosis by exposure to 50 nM T_3_ for 3 days to induce metamorphosis, or left untreated. Tadpoles were anesthetized in 0.4 mg/ml tricaine, placed in plastic petri dishes and injected with 0.1 ml of 5 µM HxBP alkyne using a 10 cc insulin needle into the tadpole head, and incubated for 30 minutes before being UV cross-linked at 100 mJ/cm^2^ and terminally anesthetized in 20 mg/ml buffered tricaine. Tails were dissected off, weighed and homogenized with a dounce homogenizer in 1 ml PBS with 0.5x EDTA-free protease inhibitor cocktail (Sigma-Aldrich).

### Isolation of Biotinylated Proteins with Avidin-affinity Beads

Samples were spun to remove insoluble organic matter and the supernatant was collected. 5 mM Rhodamine-biotin azide was covalently linked to the alkyne moiety of the HxBP probe using click chemistry [26] in the presence of 50 mM *tris*(2-carboxyethyl)phosphine (TCEP), 50 mM CuSO_4_ and 1.7 mM *tris*[(1-benzyl-1H-1,2,3-triazol-4-yl)methyl]amine dissolved in DMSO and *t*-butanol at a ratio of 1∶4. Unreacted azide was removed using 5 ml Zeba-Spin desalting columns (Pierce) and the labeled mixture diluted in PBS with 0.5% SDS. Avidin-agarose beads were pre-washed in PBS and incubated overnight at 4°C with the labeled homogenate to isolate biotinylated proteins. Beads were then washed once with 0.2% SDS in PBS, once with 6M urea and thrice with PBS to remove non-specifically bound proteins.

For samples that were to be resolved by SDS-PAGE, beads were boiled for 15 minutes in 5× reducing SDS-PAGE sample buffer, run on a 10% polyacrylamide gel for 2 hours at 120 V, and either silver stained and photographed, or blotted to a PVDF membrane. Proteins that were transferred to a PVDF membrane were blocked overnight in blocking buffer (PBSTw with 5% BSA) at 4°C, then incubated overnight at 4°C in a 1∶10000 dilution of Streptavidin-HRP. After three 10 minute washes in PBSTw, the membrane was detected using ECL (Pierce).

### Gelatin Zymography of Labeled Tadpole Homogenates

Frozen tadpole homogenates were thawed, spun and diluted 1∶4 with 5X non-reducing sample buffer (225 mM Tris pH 6.8, 50% glycerol, 5% SDS, 0.05% bromophenol blue) and left at room temperature for 5 minutes. Samples were run on a 10% polyacrylamide gel containing 0.1% bovine gelatin for 3 hours at 110 V. The gel was then washed twice for 20 minutes in renaturing buffer (1.25% Triton X-100 dissolved in 25 mM Tris pH 7.5) and twice for 20 minutes in developing buffer (50 mM Tris pH 7.5, 5 mM CaCl_2,_ 0.1 mM ZnSO_4_, 0.025% Brij35) at room temperature with gentle agitation, then placed in fresh developing buffer and incubated at 28°C for 48 hours. The gel was stained with Coomassie R-250, and then photographed once lytic bands were clearly visible.

## Results

### *HxBP* Labels active *MMP-2* in vitro

**Figure 2:**
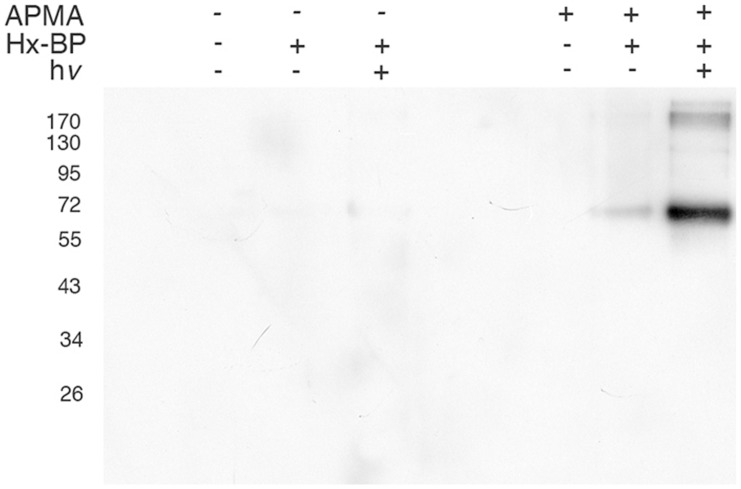
Hx-BP labels active MMPs *in vitro*. 135 ng of human recombinant MMP-2 (hrMMP-2) was incubated with 0.5 nmol of biotinylated HxBP, with or without activation by APMA and with or without UV crosslinking, then resolved by SDS-PAGE and blotted to PVDF membrane. Streptavidin-HRP detection of biotinylated hrMMP-2 is dependent on both the activation status of the protease, and on exposure to UV light (h*v*).

In order to verify that the HxBP probe is able to label MMP-2 in our hands, we incubated full-length human recombinant MMP-2 (hrMMP-2), either with or without APMA activation, with biotinylated HxBP, UV-cross-linked, then blotted and probed with streptavidin-HRP. Consistently with the results of Saghatelian *et al.* [1], biotinylation of hrMMP-2 in this experiment depended on UV-cross-linking, and the activation state of hrMMP-2 (Fig. 2). Very little of the hrMMP-2 protein is labeled when the UV-cross-linking is conducted with the prozyme form of the enzyme, suggesting that the ‘cysteine switch’ of the propeptide effectively prevents the HxBP probe from interacting with the zymogen. Background labeling (without UV-cross-linking) was minimal, suggesting this probe is predictably un-reactive in the absence of excitatory light.

### *HxBP* can be used to tag *MMP2* in vivo

It is well established that T_3_-treated *Xenopus* tadpoles dramatically up-regulate their metalloproteinases during this artificially-induced metamorphosis [21], [27]. Consistent with previous observations, we observed a profound increase in metalloproteinase activity, including abundant expression of xMMP2, by gelatin zymography in T_3_-induced tadpoles compared to their un-induced siblings (data not shown). We reasoned that this well-established developmental up-regulation of xMMP2 expression could be used as a proof-of-concept to determine if HxBP can be used to tag active MMPs *in vivo*. T_3_-induced and un-induced tadpoles were injected anteriorly with 100 µl of 5 µM alkyne HxBP probe, then allowed to recover for 30 minutes while the probe diffused through their tissues and bound target molecules. Injected larvae were then UV-photocrosslinked, and homogenates of tail tissue were subjected to click chemistry with biotin azide, which results in the HxBP-tagged proteins becoming biotinylated. Biotinylated proteins were then purified by streptavidin affinity chromatography, resolved on a 10% SDS-PAGE gel, and detected by silver staining.

**Figure 3:**
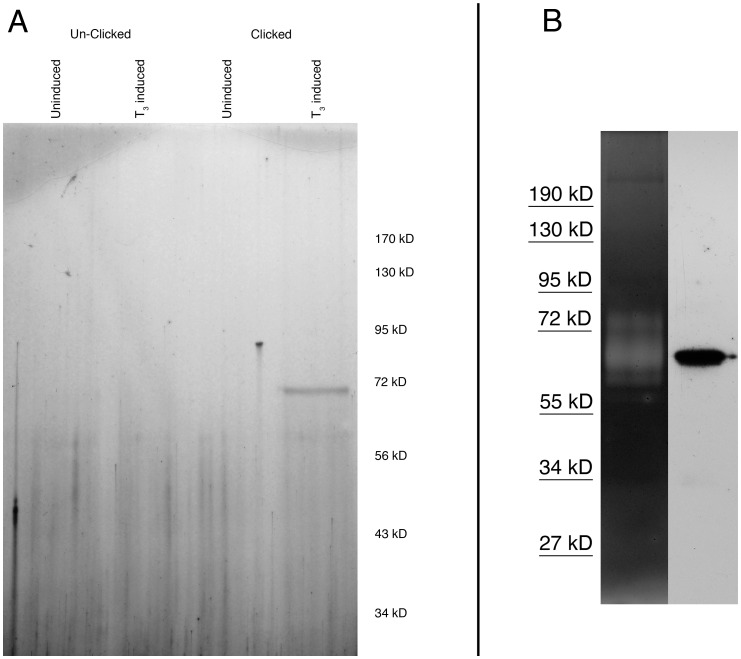
HxBP labels its target proteins *in vivo*. Live *Xenopus* tadpoles were injected with HxBP-alkyne, allowed to recover, exposed to UV light, and then sacrificed and protein extracts of the tails were subjected to click chemistry to biotinylate HxBP-tagged proteins. In panel A, biotinylated proteins were affinity purified by avidin chromatography, resolved by SDS-PAGE and detected by silver staining. A single protein with a mobility consistent with xMMP2 is detectable in the lane containing the HxBP-labeled proteome of the larvae that were induced to express xMMP2 by treatment with T3, and then biotinylated by click chemistry. In panel B, tail homogenates were split and run in parallel on a gelatin zymography gel (left) and for blotting to PVDF (right). Blots were probed with streptavidin-HRP, and revealed a single labeled protein at the same mobility as the gelatinolytic xMMP2 band on the zymograph.

A single ∼70 kD biotinylated protein is detected in the clicked proteomes of T_3_-induced tail homogenates (Fig. 3A), and we verified the identity of this protein as *Xenopus* gelatinase A (xMMP2) by gelatin zymography (Fig. 3B). Thus, active MMPs can be tagged *in vivo*, and then subsequently extracted and biochemically characterized by using the HxBP tag.

### Proteins Labeled *in vivo* Using HxBP can be Visualized in Intact Zebrafish Embryos

**Figure 4:**
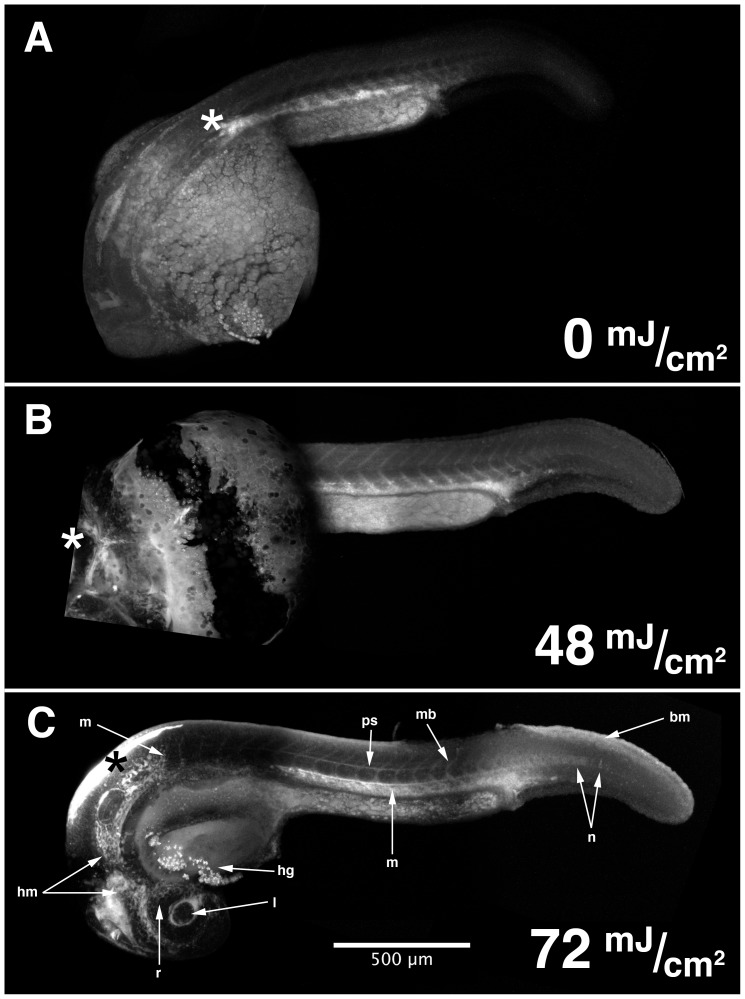
HxBP can be UV-photocrosslinked in vivo, and detected in situ by confocal microscopy. Composite confocal micrographs of 24 hpf zebrafish embryos injected anteriorly with 50 µM trifunctional HxBP probe, recovered, and exposed to increasing levels of UV irradiation reveals increasingly spatially structured patterns of fluorescence up to 72 mJ/cm^2^. Structures exhibiting strong HxBP labeling in 24 hpf embryos include the retina (r) and lens (l), head mesenchyme (hm), hatching gland (hg), perichordal sheath (ps), isolated notochord cells in the elongating region of the notochord (n), maturing myotome boundaries (mb), mesenchymal tissues of the trunk and tail (m), and the basement membrane underlying the epithelium dorsal to the elongating tail (bm). Asterisks mark the point of injection.

**Figure 5:**
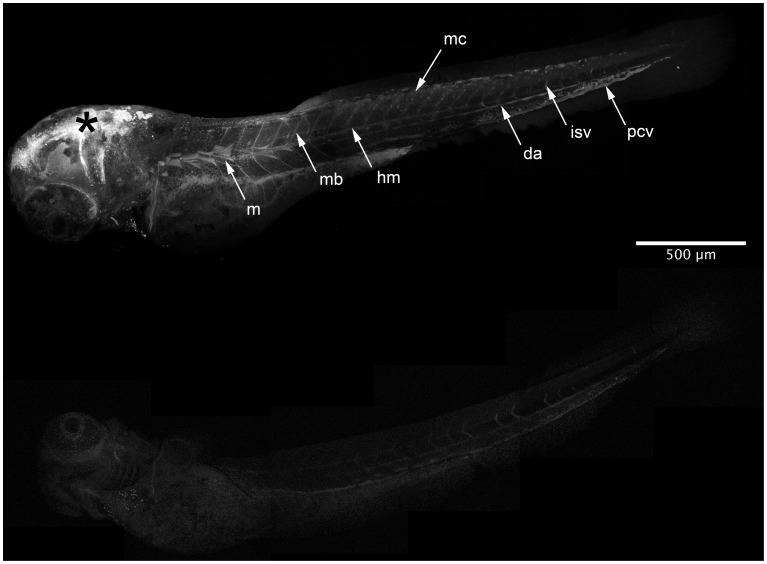
HxBP is labeling metalloproteinases *in vivo*. Composite confocal micrographs of 72 hpf zebrafish embryos injected anteriorly (*) with 50 µM of trifunctional HxBP probe either with (B) or without (A) competition from unlabeled GM6001. Embryos pre-injected with GM6001 show dramatically attenuated HxBP labeling, requiring the data shown in panel B to be collected using 42% increased gain in order to be detectable. Structures showing strong labeling in HxBP labeled 72 hpf embryos include proliferative myofibrils along the anterior lateral midline (m), maturing myotome boundaries (mb), the horizontal myoseptum (hm), individual migratory mesenchyme cells (mc), and the developing vasculature including the dorsal aorta (da), posterior cardinal vein (pcv) and intersomitic vessels (isv). Asterisk marks the injection site. Scale bar is 500 µM.

We sought to determine whether, in addition to facilitating their biochemical characterization, this activity-based probe could be used to visualize the distribution of active MMPs in living tissues. Because the optical properties of the *Xenopus* tadpole are not ideal for imaging studies, and because we have previously shown that MMP-activity can be detected using fluorogenic substrates in zebrafish embryos [18], [21], we injected rhodamated HxBP into live zebrafish embryos, and assayed its pattern of localization by confocal microscopy. In order to maximize the specificity and sensitivity of this assay, we experimented with the UV-exposure. Below 48 mJ/cm^2^, we observed weak labeling that contrasts poorly with endogenous autofluorescence in the red channel even when imaging settings are optimized to maximize signal to noise ratios (Fig. 4A). Presumably this represents residual probe interacting non-covalently with target molecules. Consistent with this, in these un-crosslinked embryos the signal is strongest in the metalloproteinase-rich hatching gland and mesenchymal tissues. Signal strength and specificity increased with UV exposure up to 72 mJ/cm^2^ (Fig. 4C), above which increased background became problematic. At 24 hpf HxBP-tagged proteins are predominantly detected in the vicinity of the lens and otic vesicle, head mesenchyme, the perichordal sheath surrounding the notochord and isolated notochord cells more posteriorly, in the hatching gland, at maturing myotome boundaries, the visceral mesoderm dorsal and posterior to the yolk extension, and in the dorsal basement membrane separating the mesoderm from the ectoderm at the elongating tip of the tail (Figs. 4 and 5). In particular the labeling in the head and visceral mesoderm, surrounding the developing lens, in the hatching gland, at the maturing myotome boundaries, in the perichordal sheath and notochord, and in the basement membrane at the dorsal posterior of the elongating tail are all sites where MMP-mediated ECM remodeling activity has been implicated or directly demonstrated previously [18], [21], [28].

To verify that the probe is interacting specifically with MMPs, we attempted to attenuate the HxBP labeling by competition with the higher affinity MMP inhibitor GM6001 (for GM6001 K_i_ = 1.1 nM, vs. 13.0 nM for HxBP against hrMMP-2 [1]). Consistent with its reported affinity for MMPs, pre-injection with GM6001 dramatically attenuated HxBP labeling in 72 hpf embryos (Fig. 5). Indeed, in order to detect any fluorescent signal in the GM6001 pre-injected embryos, the gain on the photomuliplier had to be increased by 42%, resulting in a somewhat noisy image of this control.

**Figure 6:**
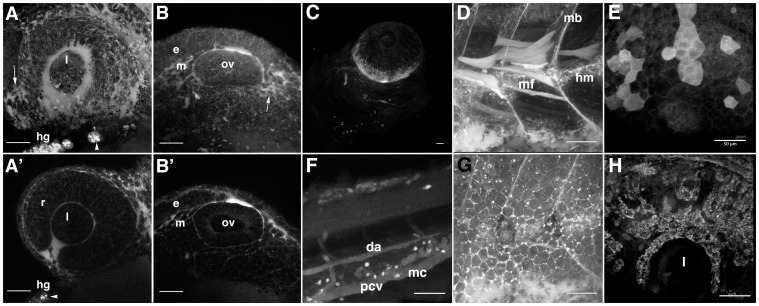
Details of HxBP labeling patterns reveal dynamics of active matrix remodeling. Z-projections of high magnification confocal micrographs illustrating patterns of HxBP labeling in both 24 hpf (A, B) and 72 hpf (C–H) zebrafish embryos. A) The developing zebrafish eye at 24 hpf, with HxBP labeling migratory mesenchyme (arrowhead), retinal epithelium (r), the choroid fissure and the hatching gland (hg). The lens shows no HxBP labeling, suggesting that matrix remodeling is absent in the lens. B) HxBP labeling the epithelial (e) and mesenchymal tissue (m) around the otic vesicle (ov), but no labeling within the otic vesicle. Primes are single focal planes of confocal stacks used to generate the non-prime panels. C) A ventrolateral view of the head of a 72 hpf embryo showing strong labeling in the developing scleral ossicles. Strong labeling is also evident in individual migratory mesenchyme cells and developing vasculature. D) lateral view of the anterior trunk showing strong labeling in the maturing myotome boundaries (mb), horizontal myoseptum (hm) and in individual myofibrils (mf) situated in the proliferative zone. E) Epithelia of the lateral head and dorsal aspect of the eye showing strong labeling in individual cells and patches of contiguous epithelial cells. F) lateral view of the tail showing strong labeling in migratory mesenchyme cells (mc), as well as labeling in the dorsal aorta (da), posterior cardinal vein (pcv) and intersomitic vessels (isv). G) lateral view of the surface epithelia covering the anterior trunk illustrating the ‘chicken wire’ patterning of HxBP labeling surrounding the periphery of the cells in this tissue. H) A dorsolateral view of the eye, illustrating strong HxBP labeling of mesenchymal cells invading across the surface of the retina and surrounding the lens (l). Scale bars are 50 µm in all panels.

In the 72 hpf embryos not pre-injected with GM6001, labeling is most notable surrounding the developing retina, in the myotomes, developing myotome boundaries and the horizontal myoseptum, in migrating meschencyme, the developing circulatory system (especially in the dorsal aorta, posterior cardinal vein and intersomitic vessels), and in the surface epithelium. High magnification images illustrating details of these and other staining patterns are shown in figure 6. Thus HxBP is an MMP-specific photo-crosslinkable reagent that can be used both to label and extract active MMPs for characterization, and to reveal their locations *in vivo*.

In the head of 24 hpf embryos, labeling is strong in the mesenchymal tissues, especially surrounding the retina and lens of the developing eye and surrounding the otic vesicle (Fig. 6, panels A and B). Individual labeled migrating mesenchyme cells can be seen in higher-magnification views (Fig. 6, panel A and B, arrows), as can the granules of the hatching gland (Fig. 6, panel A and A′, arrowheads). This latter observation is not unexpected, given that the hatching enzymes of teleost fish, including zebrafish, are well-characterized metalloproteinases [29]–[31], and it is a structure that exhibits strong activity against some (but not all) flurogenic metalloproteinase substrates [21]. Indeed, the hatching gland can essentially be considered an internal positive control in these preparations. The head of a 72 hpf embryo shows strong labeling in the developing scleral ossicles (Fig. 6, panel C), which condense from neural crest that have migrated into this location from the dorsal neural tube [32], as well as what appear to be individual neural crest cells migrating onto the pigmented retina (Fig. 6, panel H), where they give rise to iridophores [33]. Strong labeling is also evident in individual migratory mesenchyme cells elsewhere throughout the embryo (e.g. Fig. 6, panel F). The developing vasculature is also consistently labeled in HxBP probed embryos, as would be expected given the well-established importance of MMP activity in angiogenesis [34]. The anterior trunk shows strong labeling in the maturing myotome boundaries, horizontal myoseptum and either within or associated with individual myofibrils situated in the medio-lateral proliferative zone (Fig. 6, panel D). Labeling in the epithelia generally takes the form of a ‘chicken wire’ like appearance, outlining the lateral margins of cells (Fig. 6, panel G), or patches and individual cells with labeling along their basal and lateral surfaces (Fig. 6, panel E).

**Figure 7:**
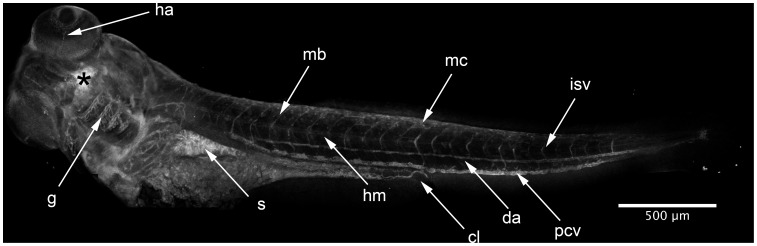
HxBP labeling in a 96 hpf swimming larva. Composite of confocal projections taken of a 96 hpf larva injected (at asterisk) with 50 µM trifunctional HxBP. Strong labeling is evident throughout the developing circulatory system, most notably in the looping vessels of the gill arches (g), the dorsal aorta (da), posterior cardinal vein (pcv), intersomitic vessels (isv), and hyaloid artery (ha). Labeling is also notable in individual migratory mesenchyme cells (mc), the protease-rich stomach (s), horizontal myoseptum (hm) and maturing craniofacial cartilages. Scale bar is 500 µm.

In 96 hpf embryos, we observe labeling in the horizontal myoseptum, myotome boundaries, cloaca, mesenchymal cells, and developing circulatory system (Fig. 7). The dorsal aorta, posterior cardinal vein, gill vasculature, intersomitic vessels, and hyaloid artery in the eye are circulatory vessels that were strongly labeled by HxBP, and known to express an abundance of Mmp9 and active Mmp25 [21], [35] (Crawford *et al.*, submitted). There is also strong labeling in the jaw cartilage and scleral ossicle, consistent with the established role for MMPs in cartilage and bone formation [36]. The labeling in the stomach is also not entirely unexpected, because of the abundance of metalloproteinases and other digestive enzymes in the gut and intestine [37].

### Identification of HxBP-tagged Metalloproteinases is not Currently Possible in Zebrafish Due to Limited Sensitivity

We were unable to extract sufficient quantities of HxBP-tagged metalloproteinases from zebrafish embryos characterize them biochemically, so we cannot unequivocally identify the proteins labeled in these samples. However, based on the results using *Xenopus* tadpoles (Fig. 3), the reported specificity of the HxBP probe for MMPs 2 and 7 [1], and the absence of an MMP-7 orthologue in the zebrafish genome [38], it seems likely that Mmp2 would be a dominant component of the labeled proteins. We are hopeful that technological advances in instrumentation and techniques will alleviate this limitation in the near future.

## Discussion

Because of their importance in development, normal physiology and disease processes, MMPs have been the focus of intense scrutiny for many years. However, because of the complexity, and largely post-translational nature of their regulation, the assays of gene expression that have proven so valuable in developing our understanding of the regulation of so many other biological processes are not as easily applied to the understanding of MMP regulation. Recent advances in the development of fluorogenic MMP substrates have been developed into methods that allow the detection of MMP activity *in vivo* [18]–[21], however these methods generate data that is difficult to quantitate and it can be complex to attribute the activity detected by these approaches to specific MMPs. Here we have used an alternative approach, involving the biochemical tagging of endogenous active protease molecules *in vivo*, rather than the detection of enzyme activity on an exogenous substrate. By subsequently purifying the tagged proteins, we were able to use *in vivo* ABPP to label xMMP2 in live metamorphosing tadpoles and subsequently purify and detect that protein on gels. In addition to providing a means of tagging and biochemically characterizing the active proteases in a living tissue, by localizing the tag *in situ*, *in vivo* ABPP allows us to determine the temporal and spatial distribution of the active proteases. Because of the 1∶1 stoichiometry of the interaction between the probe and active enzyme molecules, *in vivo* ABPP may be useful in the quantification of changes in these distributions. Moreover, by using ABPP probes in competition with putative pharmacological MMP inhibitors, this approach may allow researchers to analyze the effectiveness of novel targeting strategies *in vivo*.

Tagging molecules with HxBP-Rh *in vivo* yields striking patterns of labeling that correlate well with the patterns of MMP-dependent collagenolytic activity previously reported in zebrafish [18], and with our more recent results using synthetic fluorogenic MMP substrates [21]. *In vivo* zymography (IVZ) using either native collagen substrates or synthetic peptide substrates have shown that, in the trunk/tail of zebrafish embryos, MMP activity is most abundant in the maturing myotome boundaries, the perichordal sheath surrounding the elongating and straightening notochord, in the angiogenic ventro-lateral mesoderm caudal to the cloaca and in the basement membrane separating the somitic mesoderm from the ectoderm [18], [21]. In the head, IVZ reveals activity associated with the developing eyes, brain, otic vesicles and mesenchymal tissues [18], [21]. The patterns of labeling we observe using *in vivo* ABPP with HxBP-Rh are consistent with these reports; we see labeling surrounding the developing lens and retina (often associated with migratory mesenchyme cells), in the brain, and surrounding the otic vesicle in the head, and at myotome boundaries, associated with developing vasculature, in the perichordal sheath, and associated with migratory mesenchyme cells in the tail. For example, the HxBP labeling in the basement membrane between the epithelium and mesoderm in the dorsal portion of the tail (Fig. 4, panel C) agrees nicely with the pattern of Type IV collagen degradation observed in [18] (for example see figure 5P in that report). We also observe isolated patches and individual epithelial cells labeling with HxBP in a manner entirely consistent with our observations using synthetic peptide substrates (for example see figure 8 in [21]).

The patterns of *in vivo* ABPP labeling we observe using HxBP may also help shed light on the mechanisms of MMP activity regulation. For example, we observe individual notochord cells in the posterior (elongating) notochord labeling with HxBP (Fig. 3C). This pattern is reminiscent of the labeling patterns observed in the notochords of embryos stained with antibodies against cadherin (for example see figure 7E and F in [28]), suggesting that changes in cell-cell adhesion and/or mechanical forces acting on the cytoskeleton may play important roles in the activation of ubiquitous latent MMPs *in vivo*. This, and the previously discussed patches of epithelial activity detected by both IVZ and *in vivo* ABPP, illustrates that apparently homogeneous populations of cells can be generating very spatially discrete patterns of MMP activity and suggests that the mechanism regulating this activity has extraordinarily fine spatial resolution.

It is interesting to note that both cell-specific and interstitial labeling can be observed in these preparations, given that both membrane-bound and secreted forms of MMPs are know to be expressed in zebrafish at this time [35], [39]–[42] (Harris *et al.*, in preparation). In individual optical sections through the eye, negative stains of mesenchymal cells can be seen in the choroid fissure, and yet positively stained individual migrating mesenchyme cells can be seen in other regions of the same optical section (Fig. 6, panel A) suggesting that membrane type and secreted MMPs may be playing specific roles in the maturation of the ECM of the lens, the development of the vasculature serving the retina, and the migration of cells through the tissues surrounding the eye.

Although the axial resolution of our optical sections is not sufficient to eliminate the possibility that the HxBP labeling we observe associated with myofibrils is genuinely intracellular, there is an increasing body of evidence indicating that MMPs, and MMP-2 in particular, are both present and playing important intracellular roles [43]–[46]. Pathological activation of intracellular MMP-2 is an underlying cause of cardiac failure following ischemic reperfusion [43], but the intracellular role of Mmp2 during embryonic development has never been investigated. High-resolution micrographs of immunostains of Mmp2 show its localization to the z-disks of muscle sarcomeres (see figure 6 in [43]), consistent with high-resolution micrographs of MMP activity detected using *in vivo* ABPP (Fig. 6, panel D). Correlating the protein localization data and activity data obtained from *in vivo* ABPP suggests a possible role for MMPs in myocyte growth and proliferation, and that this intracellular pool of MMP in the myocytes is active in the medial myofibrils at this stage in development.

Combining immunostaining with *in vivo* ABPP can therefore provide a means to detect differences and similarities between MMP localization and MMP activity. Our results show that, while Mmp2 transcription can be detected abundantly throughout the embryo [39], the protein product is much more spatially heterogeneous (Harris, et al., in preparation), and its activity is yet more spatially restricted, further supporting a model whereby MMP activity is regulated by a combination of spatially heterogeneous inhibition [18] and post-translational mechanisms that concentrate these ECM remodeling effectors in specific locations. Elucidation of these mechanisms will doubtlessly provide valuable insights into the regulation of matrix remodeling in both developmental and pathological contexts.
